# Long Non Coding RNA H19: A New Player in Hypoxia-Induced Multiple Myeloma Cell Dissemination

**DOI:** 10.3390/ijms20040801

**Published:** 2019-02-13

**Authors:** Chiara Corrado, Viviana Costa, Gianluca Giavaresi, Annalisa Calabrese, Alice Conigliaro, Riccardo Alessandro

**Affiliations:** 1 Department of BioMedicine, Neurosciences and Advanced Diagnostics (Bi.N.D), Via Divisi 83, 90133 Palermo, Italy; chiara.corrado@unipa.it (C.C.); annalisacalabrese84@gmail.com (A.C.); 2 IRCCS ISTITUTO ORTOPEDICO RIZZOLI, 40138 Bologna, Italy; viviana.costa@ior.it (V.C.); gianluca.giavaresi@ior.it (G.G.); 3 Laboratory of Preclinical and Surgical Studies, IRCCS ISTITUTO ORTOPEDICO RIZZOLI, 40138 Bologna, Italy

**Keywords:** long non-coding RNA H19 (lncH19), hypoxia, multiple myeloma, HIF-1α

## Abstract

The long non-coding RNA H19 (lncH19) is broadly transcribed in the first stage of development and silenced in most cells of an adult organism; it appears again in several tumors where, through different molecular mediators, promotes cell proliferation, motility and metastases. LncH19 has been associated with hypoxia-inducible factor 1-alpha (HIF-1α) activation and, in some tumors, it has proved to be necessary and required to sustain hypoxic responses. Here we propose to investigate a putative role for the lncH19 in hypoxia induced multiple myeloma (MM) progression. Transcriptional analysis of MM cell lines (RPMI and MM1.S) exposed to normoxia or hypoxia (1% O_2_) was done in order to evaluate lncH19 levels under hypoxic stimulation. Then, to investigate the role of lncH19 in hypoxia mediated MM progression, transcriptional, protein and functional assays have been performed on hypoxia stimulated MM cell lines, silenced or not for lncH19. Our data demonstrated that hypoxic stimulation in MM cell lines induced the overexpression of lncH19, which, in turn, is required for the expression of the hypoxia induced genes involved in MM dissemination, such as C-X-C Motif Chemokine Receptor 4 (CXCR4) and Snail. Moreover, adhesion assays demonstrated that lncH19 silencing abrogates the increased adhesion on stromal cells induced by the hypoxic condition. Finally, Western blot analysis indicated that lncH19 silencing impaired HIF1α nuclear translocation. The LncH19, required for the induction of hypoxic responses in MM cells, could represent a new therapeutic target for MM.

## 1. Introduction

Increasing evidence has demonstrated the involvement of several long non-coding RNAs (lncRNAs) in the onset and progression of different neoplasms, including multiple myeloma (MM) [1,2] so that, most of them, including lncH19, are now recognized as oncogenes [3]. LncH19 was among the first lncRNAs identified; it was originally studied for its involvement in embryonic development and, today, it is considered one of the major players in cancer, controlling proliferation, differentiation and cell motility [4,5]. LncH19 can control gene expression through several mechanisms of action; it is involved in the supervision of DNA methylation [6], as well as in the regulation of intracellular miRNA pattern, being both a sponge for miRNAs and a micro RNA reservoir [7,8]. LncH19 exon one encodes for two different miRNAs, miR-675-3p and miR-675-5p, and through them, it controls most of the tumor processes in which it is involved [9,10,11,12,13].

Concerning the mechanism driving lncH19 expression, among the proposed transcription factors, recent evidence formally demonstrated that the hypoxia master regulator HIF-1α directly binds the lncH19-promoter, thus inducing its transcription [14].

A hypoxic microenvironment is a common feature of tumors; it is closely correlated to tumor progression and negatively affects clinical outcome by promoting genetic instability, tumor cell metastasis and invasiveness [15]. A family of transcription factors called hypoxia-inducible factors (HIF-1α and HIF-2α) mediates these effects, in particular HIF-1α activity which is highly dependent on oxygen supply. In normoxic conditions, HIF-1α is rapidly hydroxylated by prolyl hydroxylase domain (PHD)-containing proteins and, subsequently, poly-ubiquitinated by the von Hippel–Lindau tumor suppressor (pVHL) for proteasome-mediated degradation. Under hypoxia, pVHL-mediated HIF-1α degradation is abolished, causing HIF-1α accumulation and nuclear translocation. Here, after the interaction with HIF-1β, the dimer binds hypoxia-responsive elements (HREs), thus inducing the transcription of numerous genes including transcription factors, histone modifiers, cytokines, surface molecules, membrane proteins and non-coding RNAs. These hypoxia-induced genes, once coordinately expressed, strongly modify the tumor cell, thus promoting a more aggressive phenotype.

The molecular mechanisms by which hypoxia controls tumor progression has been largely dissected, while less information is available about this aspect in hematological disease, although the contribution of HIFs is now consolidated in this context [16,17].

Multiple myeloma (MM) is the second most common hematological malignancy and it affects plasma cells (PCs) in the bone marrow (BM). The pathology, starting with asymptomatic monoclonal gammopathy, could evolve to malignant disease with end-organ damage, renal insufficiency, anemia, increased BM angiogenesis and osteolytic bone lesions associated with significant patient morbidity [18]. Hypoxia has been found to stimulate MM progression by enforcing cancer stem cell population [19], as well as by increasing the migration and homing of circulating MM cells to new BM niches. Low O_2_ partial pressure induces, in MM cells, a transformation similar to an epithelial to mesenchymal transition (EMT) that promotes CXCR4 expression and prompts cells to move to the metastatic site [20].

Recently, aberrant expression of lncH19 has been associated with circulating IL-6 or IL-8 levels and poor prognosis for MM patients [21]; moreover, Pan et al. proposed the upregulation of serum lncRNAH19 as a novel biomarker for early diagnosis and clinical treatment of MM [22]. However further data are required to clarify the role of lncH19 in MM.

Here we investigated the correlation between the lncH19 aberrant expression and hypoxic condition in MM cell lines.

## 2. Results

### 2.1. Hypoxic Stimulation Induced LncH19 Overexpression in MM Cell Models

**Figure A1:**
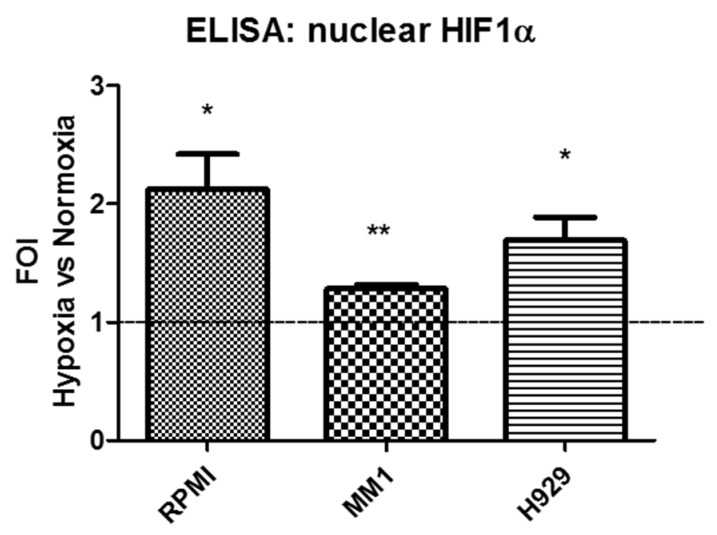
ELISA assay for nuclear HIF1-α indicates that three different multiple myeloma cell lines respond to 24-h hypoxic chamber stimulation. Values are presented as mean ± SD. Statistical analysis was performed by Student-t test: * *p* < 0.05; ** *p* < 0.01.

In order to evaluate the effects of hypoxia on MM cells, we made use of three different cellular models: MM1.S, RPMI and H929 cells. The hypoxia responsiveness of all cell lines was tested after a 24-h incubation period in a hypoxic chamber. As shown in Appendix A Figure A1, all MM cells respond to hypoxic stimulation by increasing HIF-1α nuclear levels, as demonstrated by ELISA assay from nuclear extracts.

**Figure 1:**
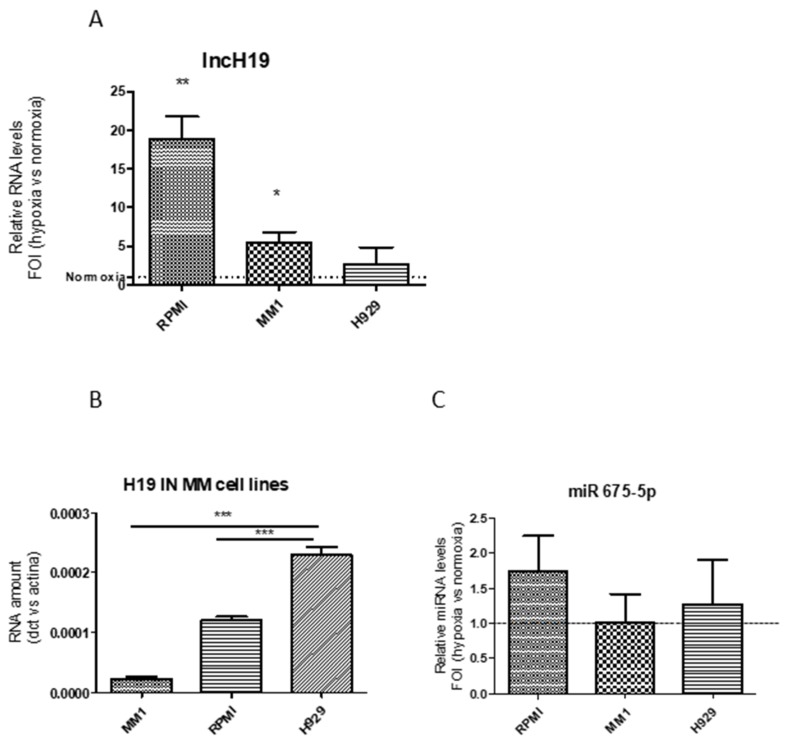
qRT-PCR indicate long non coding H19 (lncH19) levels in Multiple Myeloma (MM) cell lines after 24-h hypoxic stimulation expressed as fold of induction versus lncH19 levels in normoxia. Statistical analysis was performed by the use of student t-test; * *p* < 0.05; ** *p* < 0.01 (**A**). qRT-PCR indicate the basal level of the lncH19 in normoxic MM cell lines. Statistical analysis was performed by the use of one way ANOVA test and Dunnett’s multiple comparison test; *** *p* = 0.001 (**B**). qRT-PCR indicate the levels of miR-675-5p in MM cell lines after 24-h hypoxic stimulation expressed as fold of induction versus normoxia (**C**). Values are presented as mean ± SD.

First, we evaluated the effects of hypoxia on lncH19 expression. The qRT-PCR in Figure 1A indicated that, in MM1.S and RPMI cell lines, lncH19 expression increased after hypoxic stimulation while an insignificant increase was found in H929 cells. This could be probably due to the higher basal level of the lncH19 in normoxic H929 cells compared to other cell lines (Figure 1B).

Previous data obtained from two different solid tumors revealed that lncH19, induced by hypoxic stimulation, sustains hypoxic responses through the selective upregulation of one of its intragenic miR-675-5p [12,13]. Surprisingly, the qRT-PCR in Figure 1C showed that lncH19 upregulation was not associated with miR-675-5p overexpression in MM.

### 2.2. LncH19 Sustained Hypoxic Response in MM Cell Lines

**Figure 2:**
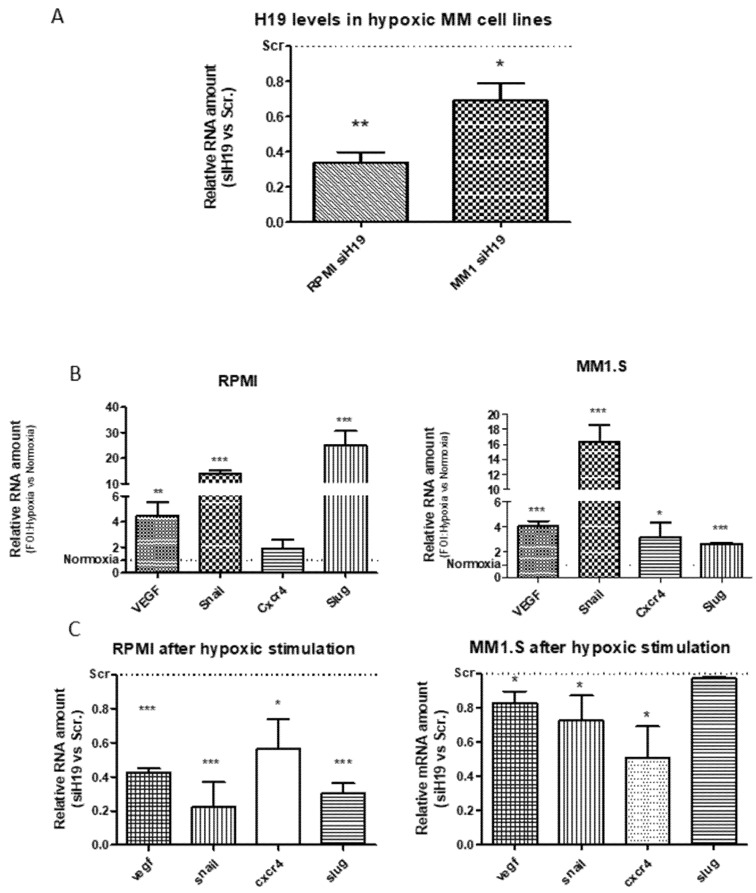
qRT-PCR indicate the H19 expression levels after hypoxic stimulation in MM cell lines infected with siH19 and relative controls. Value are expressed as Fold Of Increase (FOI) respect to siRNA Scramble (siScr) infected cells (**A**). qRT-PCR of indicated genes in MM cell lines after hypoxic stimulation compared to normoxia. Value are expressed as FOI respect to normoxic cells (**B**). qRT-PCR of indicated genes in hypoxic MM cell lines silenced or not for lncH19. Value are expressed as FOI respect to siScr infected cells (**C**). Values are presented as mean ± SD. Statistical analysis was performed by the use of Student *t*-test. * *p* < 0.05; ** *p* < 0.001; *** *p* < 0.0001.

With the aim to investigate a direct role of lncH19 in hypoxic responses, we subjected MM cell lines stably silenced for H19 (siH19) and relative control cells (siScr) to hypoxic stimulation. After observing that lncH19 expression did not significantly increase in H929 cells after hypoxic stimulation, we decided to perform the subsequent experiments on the other two cell lines, RPMI and MM1.S. qRT-PCR analysis in Figure 2A show H19 silencing efficiency in MM cell lines after hypoxic stimulation. In order to investigate the effects of siH19 on hypoxic responses, transcriptional analysis was done on the HIF targets known to be involved in tumor progression and multiple myeloma dissemination: Vascular Endothelial Growth Factor (VEGF), C-X-C chemokine receptor type 4 (CXCR4) and the transcription factors Snail and Slug [19,20,23]. As expected, HIF targets are upregulated after hypoxic stimulation (Figure 2B) while, surprisingly, this overexpression is impeded by lncH19 silencing in both cell lines (Figure 2C). These data indicated that the lncH19 expression is required for the HIF-induced MM dissemination.

### 2.3. H19 Silencing Affected the Hypoxia-Induced Adhesion of MM Cells on the Stroma

**Figure 3:**
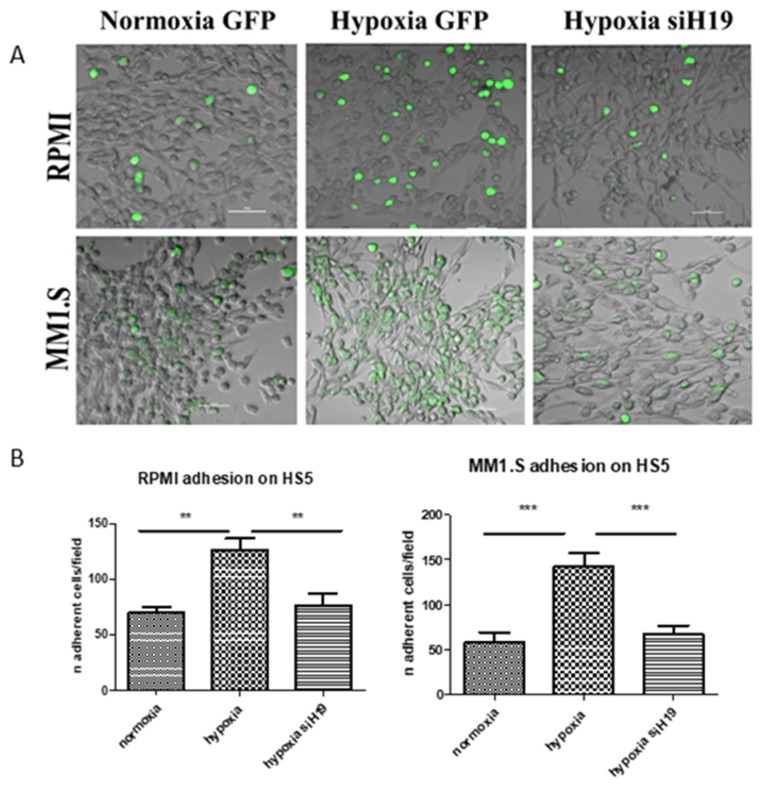
Adhesion assay of MM cells to stromal monolayer, in normoxia and after hypoxic stimulation, silenced or not for lncH19. Representative images of different experimental condition captured by Nikon A1 confocal microscope, scale bar = 50 μm (**A**); quantification of Green Fluorescent Protein (GFP) positive MM adherent cells. Values are presented as mean ± SD. Statistical analysis was performed by the use of one way ANOVA test and Dunnett’s multiple comparison test ** *p* < 0.001; *** *p* < 0.0001 (**B**).

In MM, hypoxia-induced CXCR4 expression promotes metastases, enhancing chemotaxis to SDF-1α and adhesion to bone marrow stromal cells [21]. In line with this evidence, and considering the inhibitory effects of lncH19 silencing on hypoxia induced metastatic genes, we evaluated the effects of lncH19 silencing on the ability of MM cells to adhere to the stromal monolayer. As shown by confocal microscopy images, both MM cell lines, stimulated by low O_2_% condition, increased their ability to adhere to stromal cell monolayer while this property is strongly inhibited by lncH19 silencing (Figure 3A). These results suggest for the first time the use of lncH19 silencing as a possible strategy to inhibit MM cell adhesion to stromal monolayer.

### 2.4. LncH19 Promoted HIF-1α Activation in Hypoxic MM Cells

**Figure 4:**
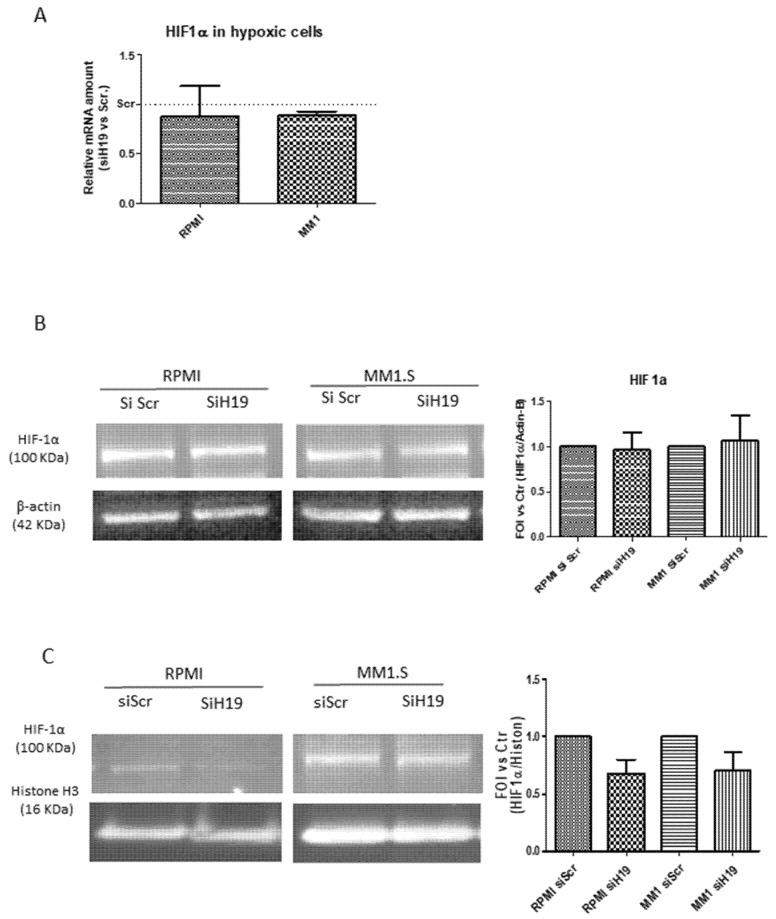
qRT-PCR (**A**) and Western blot analysis on total protein extract (**B**) of HIF-1α expression in MM cells silenced or not for lncH19. Densitometric analysis with Image J software was done with respect to total protein level of β-actin, used as loading control. (**C**) Western blot analysis on nuclear extract of MM hypoxic cells silenced or not for lncH19. Densitometric analysis with Image J software was done with respect to nuclear protein level of histone H3, used as loading control. Values are presented as mean ± SD.

In order to investigate a putative molecular mechanism by which lncH19 could affect hypoxic responses, we analyzed the effects of lncH19 silencing on HIF-1α. As shown in Figure 4, siH19 affected neither the mRNA levels of the transcription factor nor the protein level of total extract in hypoxic MM cells (Figure 4A,B). Interestingly, as revealed by Western blot analysis on nuclear extracts, H19 silencing reduced the HIF-1α nuclear translocation in hypoxic condition (Figure 4C), thus suggesting for the first time a role of lncH19 in HIF-1α activation.

**Figure 5:**
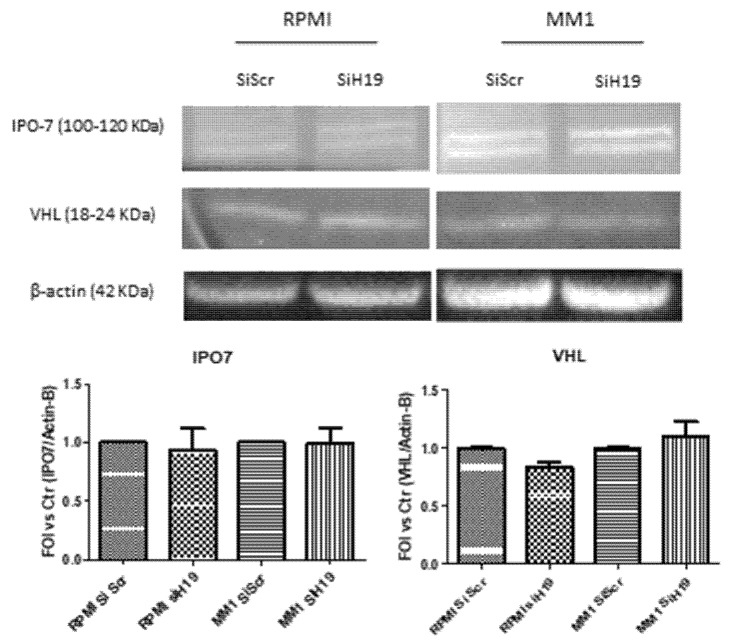
Western blot analysis of IPO7 and VHL on total extract of MM hypoxic cells silenced, or not, for lncH19. Densitometric analysis with Image J software was done with respect to total protein level of β-actin, used as loading control. Values are presented as mean ± SD.

With the aim to further investigate the molecular mediator by which lncH19 modulate HIF-1α nuclear translocation, we analyzed two different proteins involved in HIF-1α stability and activation: VHL, the E3 ubiquitin ligase responsible of HIF-1α cytoplasmic degradation, and the importin IPO7 that is directly involved in HIF-1α nuclear translocation [24]. As shown by the Western blot analysis (Figure 5) the lncH19 silencing did not affect the protein levels of VHL either of IPO7. Further studies are required to identify the factors that, regulated by lncH19, could control HIF-1α activation and nuclear translocation.

## 3. Discussion

BM niche has a central role in the onset and progression of hematologic malignancies. The BM is physiologically hypoxic and the low O_2_ partial pressure guarantees initial MM cell survival and growth. Disease progression is associated with the expansion of hypoxic niches and stabilization of the oncogenic HIF-1α; activation of the HIF-1α pathway in growing MM cells gives them the input to disseminate into new BM sites [25]. First, Azab et al., formally demonstrated that hypoxia is the driving force to support MM metastasis through both i) the induction of an EMT like phenotype, that promotes MM cell motility, and ii) the up regulation of CXCR-4 that permits homing of circulating MM cells to new BM niches [21]. Recent evidence shows that the hypoxia master regulator HIF-1α can induce lncH19 transcription in glioblastoma [14]. Related to MM, Sun et al. provided evidence, in vitro and in vivo, that abnormal upregulation of lncH19 is correlated to MM progression and its expression in bone marrow is associated with poor prognosis for patients [21]. Most recently, Pan et al. enforced the association between MM and lncH19 demonstrating a positive correlation between the progression of the hematological disorder and its serum levels [22]. This study provides, to our knowledge, evidence of a direct correlation between HIF-1α, the master regulator of hypoxic response, and the lncH19 in MM. We demonstrated in two different multiple myeloma cell lines that lncH19 overexpression is induced by hypoxic stimulation and that its overexpression is functional for the hypoxia- induced MM dissemination.

In our previous study, we demonstrated that lncH19 controls the hypoxia-induced EMT in colon cancer cell through its intragenic miR-675-5p and that, overexpressed after hypoxic stimulation, targeted Snail inhibitor DDB2 [12]. Moreover, also in a glioblastoma model, we demonstrated that lncH19 controls both expression and activity of HIF-1a through miR-675 [11].

Here, MM presents a different scenario for the HIF-1α/lncH19 interaction. Induced by HIF-1a, the lncH19, instead of being processed for its miRNA maturation, directly participates in hypoxia-induced EMT phenotype. The expression of Snail and Slug are pivotal events for the induction of EMT; the lncH19 silencing abrogated the HIF-induced expression of the two master genes blocking the downstream transformation. The inhibitory effects induced by lncH19 silencing are enforced also by the downregulation of another two HIF-1α targets with an essential role in MM progression: CXCR-4 and VEGF. CXCR-4/CXCL12 signaling, crucial in the homeostasis of the adult hematopoietic system [26,27], is involved in driving cell motility of MM cells from primary hypoxic site to a new area of implant osteolytic lesion [28]. Moreover, VEGF, through the stimulation of vascular permeability and endothelial cell migration, is among the most important cytokine involved in the stimulation of bone marrow microvessel density (MVD) in MM progression [28]. Angiogenesis is an attractive target for MM therapy. Antiangiogenic strategies are commonly used to treat neoplasms such as VEGF neutralizing antibody (Avastin^®^), immunomodulatory drugs (IMiDs) [29] or the proteasome inhibitors, bortezomib and carfilzomib [30,31]. Alternative anti-angiogenetic strategies are already under investigation; recently, Rao and collaborators described a new drug, MP0250, that is able to bind and neutralize both VEGF and HGF, thus making an anti-angiogenic effect on MM in vivo [32].

Related to the molecular mechanisms interplaying between the lncH19 and HIF-1α, we demonstrated that lncH19 silencing did not affect either HIF-1α transcript or its protein levels. However, further investigation suggested a putative role of lncH19 in the nuclear accumulation of activated HIF-1α. This, to our knowledge, is the first evidence that correlates a lncRNA to HIF-1 α activation. HIF-1α is shuttled from cytosol to nuclei and this is determinant for its activity; it was recently demonstrated that HIF-1α nuclear translocation is driven by importins 4 and 7, with a specific physical interaction between HIF-1α and importin 7 (IPO7) [24]. Furthermore, our group previously demonstrated that IPO7 is responsible for HIF-1α nuclear translocation in chronic myelogenous leukemia cells [33].

However, HIF-1α nuclear accumulation is a complex process and several mediators could contribute to this process. Our data lead us to exclude a role for both IPO7 and VHL, in the H19/HIF-1α cross-talk; further studies need to be performed to identify the players involved in the regulation of HIF-1α localization induced by lncH19.

Today, although hypoxia inhibitors, such as Bortezomib and Lenalidomide, are proposed in MM treatment, we lack an efficient instrument to block MM cells dissemination, osteolysis and angiogenesis in myeloma patients. The identification of the factor(s) involved in the recirculation and dissemination process in MM is key in the development of therapeutic strategies.

Our studies suggest the lncH19 as a putative therapeutic target for the treatment of hypoxia-induced MM cells dissemination.

## 4. Materials and Methods

### 4.1. Cell Culture and Reagents

Human MM cell lines (RPMI, H929, and MM1.S) and Human stromal cells (HS5) were obtained from American Type Culture Collection (ATCC, LGC standards, Milan, Italy). All MM cells were routinely maintained in RPMI-1640 medium (Euroclone s.p.a., Milan, Italy); HS5 were maintained in DMEM medium (ATCC) and supplemented with 10% fetal bovine serum (FBS) and 1% Pen/strep (all from Euroclone s.p.a. Cells were maintained in a 37 °C humidified atmosphere of 5% CO_2_. To induce hypoxic stimulation, MM cell lines were seeded at 500,000 cells/ml and incubated in a “Hypoxic Chamber”containing 1% O_2_ gas mixture for 24 h. After hypoxia stimulation, cells were immediately kept on ice and processed.

### 4.2. Cell Infection

Human MM cell lines were seeded at 400,000 cells in 1 ml of RPMI 1640 complete medium, 45 µl of lentivirus (piLenti-siH19-GFP lentivirus or its control, piLenti-GFP lentivirus) with a titer of 1 × 10^8^ UI and 10 µg of Polybrene^®^ (Santa Cruz Biotechnology Inc., Heidelberg, Germany) were added. After 24 h, the medium was replaced in order to remove the lentiviruses and fresh complete medium was added. In order to select and stabilize lentivirus-infected cells containing puromycin resistant selection marker, cells were maintained with 0.3 µg/ml puromycin (Sigma Aldrich SRL, Milan, Italy).

### 4.3. RNA Extraction and Real-Time PCR

Total RNA was extracted using the commercially available illustraRNAspin Mini Isolation Kit (GE Healthcare, Milan, Italy), according to the manufacturer’s instructions. RNA was reverse transcribed to cDNA using the High Capacity cDNA Reverse Transcription Kit (Applied Biosystems, ThermoFisher Scientific, Monza, Italy). Quantitative RT-PCR (qRT-PCR) analysis was performed in duplicates for each data point, using custom made primers (Invitrogen, Life Technologies, Monza, Italy) as described in Table 1. The mean threshold cycle was used for the calculation of relative expression using the Livak method against ACTB as the reference gene. For miRNA expression, 250 ng of RNA was reverse transcripted according to the manufacturer’s instructions (cat. number 4366596, TaqMan MicroRNA Reverse Transcription, Applied Biosystems). TaqMan probes were used to analyze: miR675-5p (cat. number 4440887, Applied Biosystems), U6 (cat. number 4427975 Applied Biosystems), Actin-β (Hs01060665_g1Applied Biosystems) and H19 (Hs00262142_g1 Life Technologies). Changes in the target miRNA content relative to housekeeping U6 were determined with the ΔΔ*C*t method.

### 4.4. Nuclear Protein Extraction and ELISA

An ELISA-based kit (TransAM Kit, Vinci-Biochem, Vinci (Firenze), Italy) was used to detect and quantify HIF-1α transcriptional factor activity following the manufacturer’s instructions. Briefly, nuclear extracts were first prepared using the Nuclear Extract Kit (Vinci-Biochem). A total of 8–10 µg of the samples were added to the coated plate and analyzed at 450 nm with Gen5 Microplate Collection & Analysis Software Data (BioTek Instruments, Bad Friedrichshall, Germany). Data were expressed as HIF-1α protein content in total nuclear extract (Absorbance) or in terms of FOI compared to control cells.

### 4.5. Adhesion Assay

In order to evaluate the ability of RPMI and MM1 untreated and H19-silencing cells to adhere to human stromal cells (HS5), an adhesion assay was performed. Briefly, HS5 monolayer, previously fixed with glutaraldehyde, was washed with PBS and incubated for 3 h with MM cells infected with different lentivirus (piLenti-siH19-GFP lentivirus and its control piLenti-GFP lentivirus), previously stimulated by 24 h hypoxic condition or not. Nikon Confocal A1 and Nis Analysis software were used respectively to reveal and count GFP positive adherent cells. Each test group was assayed in triplicate; five fields were counted for each condition.

### 4.6. Total Protein Extract and Western Blot Analysis

MM cells after wash in PBS were lysed in lysis buffer (300 mM NaCl, 50 mM Tris HCl pH 7.6, 0.1% Triton X-100, 1 mM PMSF, 10 μg/ml leupeptin, 10 μg/ml aprotinin, 4 mM EDTA, phosphatase inhibitors). After 1.5 h of incubation on ice, total cell lysates were clarified using high-speed centrifugation for 15 min and an aliquot of the supernatant was assayed to determine protein concentration by Coomassie plus protein assay reagent (Thermo Fisher Scientific, Rockford, IL, USA). Proteins were separated by SDS-polyacrylamide gel electrophoresis (Bolt Bis-Tris gel 4–12%, Thermo Fisher Scientific), and transferred to nitrocellulose membrane (GE Healthcare, Milan, Italy). The membrane was incubated in blocking solution (5% non-fat dry milk, 20 mM Tris, 140 mM NaCl, 0.1% Tween-20), and probed overnight at 4 °C with specific antibodies against HIF1α (Millipore) and IPO7 (Pierce, Thermo Fisher Scientific); Histone H3 and VHL (Santa Cruz Biotechnology, SantaCruz, CA, USA); β-actin (Cell Signalling Technology, Beverly, MA, USA). After three washes with 20 mM Tris, 140 mM NaCl, 0.1% Tween-20, the membrane was incubated 1 h with secondary antibody daylight 488 (Thermo Fisher Scientific) diluted in blocking solution. After signal detection by Chemidoc (Biorad, Milan, Italy), densitometric analysis was done with Image J software (National Institute of Health, Bethesda, MA, USA).

### 4.7. Statistical Analysis

Statistically significant differences between mean values (from at least three independent experiments) were determined using one-tailed T Student’s test. Differences were considered statistically significant at *p* < 0.05. Other sets of experiments were evaluated with one-way ANOVA analysis and Dunnett’s multiple comparison test, as described in the figure legends.

## Figures and Tables

**Table 1:** Primers list.

*Gene*	*Primer forward*	*Primer Reverse*
**LncH19**	GCACCTTGGACATCTGGAGT	TTCTTTCCAGCCCTAGCTCA
**HIF-1α**	TGATTGCATCTCCATCTCCTACC	GACTCAAAGCGACAGATAACACG
**VEGF**	CGAGGGCCTGGAGTGTGT	CGCATAATCTGCATGGTGATG
**SNAIL**	GCGAGCTGCAGGACTCTAAT	CCCGCAATGGTCCACAAAAC
**SLUG**	CATGCCTGTCATACCACAAC	GGTGTCAGATGGAGGAGGG
**CXCR4**	TACACCGAGGAAATGGGCTCA	AGATGATGGAGTAGATGGTGG
**B−A TIN**	ATCAAGATCATTGCTCCTCCTGA	CTGCTTGCTGATCCACATCTG
